# Differential Effects of Parkinson’s Disease on Interneuron Subtypes within the Human Anterior Olfactory Nucleus

**DOI:** 10.3389/fnana.2017.00113

**Published:** 2017-12-05

**Authors:** Isabel Ubeda-Bañon, Alicia Flores-Cuadrado, Daniel Saiz-Sanchez, Alino Martinez-Marcos

**Affiliations:** Neuroplasticity and Neurodegeneration Laboratory, CRIB, Ciudad Real Medical School, University of Castilla-La Mancha, Ciudad Real, Spain

**Keywords:** calcium binding protein, non-motor symptoms, olfaction, somatostatin, α-synucleinopathy

## Abstract

Synucleinopathies (including α-synucleinopathies), which include Parkinson’s disease (PD), manifest themsevles early on (stage 1) in the olfactory system; preferentially in the anterior olfactory nucleus (AON). In particular, the non-motor, early manifestations of PD include hyposmia, which is the partial loss of the sense of smell. The neural basis of hyposmia in PD, however, is poorly understood; but the AON appears to be a key structure in the disease’s progression. We analyzed whether α-synuclein was involved in the differential interneuron vulnerability associated with PD in the retrobulbar, cortical anterior and cortical posterior divisions of the AON. First, we determined the expression of the calcium binding interneuron markers, calretinin, calbindin and parvalbumin, as well as non-calcium binding interneuron marker, somatostatin, in neuronal cell bodies alone (cells/mm^2^) and in neuronal cell bodies and neurites (% of area fraction) of post-mortem tissue from PD cases and age-matched controls (*n* = 4 for each) by immunofluorescent confocal microscopy. Results indicated that parvalbumin expression was upregulated in neuronal cell bodies throughout the anterior olfactory nucleus of PD cases compared with controls. Furthermore, there was increased calbindin, calretinin and parvalbumin expression in the cell bodies and neurites of neurons in the retrobulbar division and also increased parvalbumin expression in the neurites of neurons in the cortical division; calretinin expression was also increased in neuronal cell bodies and neurites in the cortical posterior division. Second, we analyzed the co-localization of the above markers with α-synuclein, with results indicating that α-synuclein co-localized with the calcium-binding proteins, but only partially with somatostatin. Taken together, these results indicate differential expression levels among different neural markers in the divisions of the AON in PD cases and point to several possibilities, among them: possible neuroprotective mechanisms of calcium-binding proteins against α-synuclein; and the differential involvement of somatostatin in α-synuclein-positive cell bodies and neurites.

## Introduction

The classical motor dysfunctions of Parkinson’s disease (PD) are bradykinesia, rigidity, resting tremor and postural instability (Lees et al., 2009; Kalia and Lang, 2015; Poewe et al., 2017); while early non-motor manifestations include dysautonomia, rapid eye movement (REM) sleep disorder and hyposmia (Stiasny-Kolster et al., 2005; Berg et al., 2015; Postuma et al., 2015; Schapira et al., 2017). In parallel with the occurrence of non-motor and motor signs, neuropathological aggregates of α-synuclein and ubiquitin, termed Lewy bodies and Lewy neurites (Spillantini et al., 1997; Goedert et al., 2013; Kalia and Kalia, 2015) appear in a predictable and cumulative six-stage sequence.

Two decades after the presence of α-synuclein in Lewy bodies was demonstrated (Spillantini et al., 1997), the precise mechanism of α-synucleinopathy in neurodegeneration remains unknown. Research in this area has revealed the following: (1) a prodromal period characterized by non-motor manifestations such as olfactory degeneration (hypnosmia), REM sleep disorder and constipation (Postuma and Berg, 2016; Sauerbier et al., 2016; Schapira et al., 2017); (2) aggregation of α-synuclein has been used to establish a cumulative and predictable sequence of six neuropathological stages in PD (Del Tredici et al., 2002; Braak et al., 2003a,b; Del Tredici and Braak, 2016; Braak and Del Tredici, 2017). In stage I, the enteric nervous system—Meissner’s and Auerbach’s plexuses and the dorsal motor nucleus of the vagus nerve (Klingelhoefer and Reichmann, 2015)—and the olfactory system—the olfactory mucosa (Saito et al., 2016), the olfactory bulb (OB) and portions of the anterior olfactory nucleus (AON)—are involved. In stage II, α-synucleinopathy progresses to the brainstem to reach the substantia nigra (stage III): the stage where motor symptoms appear and clinical diagnosis can be established (Del Tredici and Braak, 2016). Aggregation of α-synuclein in the central and peripheral nervous system structures has been correlated with both non-motor and motor symptoms (Tolosa and Pont-Sunyer, 2011; Salat et al., 2016), but a direct clinicopathologic relationship remains controversial (Burke et al., 2008). (3) In addition, the progression of α-synucleinopathies is still a matter of current debate. Interestingly, there is accumulating evidence that α-synucleinopathies associated with neurodegenerative diseases can spread via neuronal pathways in a prion-like misfolding and seeding aggregation manner (Jucker and Walker, 2013; Walker and Jucker, 2015). Furthermore, specific α-synuclein conformations may be particularly toxic and relevant to non-motor manifestations without contributing to motor symptoms (Kalia and Kalia, 2015).

Although neural substrates of hyposmia in PD have not been identified (Doty, 2012a,b, 2017), initial entry sites of α-synucleinopathies (Kalia and Kalia, 2015) include the enteric nervous system and the olfactory system (Braak et al., 2003a; Del Tredici and Braak, 2016; Braak and Del Tredici, 2017). Specifically, different divisions of the AON are preferentially involved (Hawkes and Shephard, 1993; Pearce et al., 1995; Braak et al., 2003a; Ubeda-Bañon et al., 2010, 2014; Attems et al., 2014; Del Tredici and Braak, 2016; Braak and Del Tredici, 2017).

To this end, we hypotheisize that the human AON is an early and preferential region of initial α-synucleinopathy (Attems et al., 2014) in PD for several reasons: its peripheral location; and also its multiple short and long distance, centrifugal and centripetal, and bilateral neural connections (Ubeda-Bañon et al., 2014). Analysis of the AON of postmortem PD patients can provide insight into the involvement and differential vulnerability of the various neuronal populations in this region. Previous reports have shown that in the olfactory system of PD patients, calcium-binding protein-expressing neurons are highly co-localized with α-synuclein, which is rarely the case for tyrosine hydroxylase- or somatostatin-positive cells (Sengoku et al., 2008; Ubeda-Bañon et al., 2010). However, comparisons on differential neural vulnerability between PD and control cases in these structures have not been carried out.

In the present report, we compared the expression of calcium-binding proteins and somatostatin in the retrobulbar and cortical anterior and poster divisions of the AON (the anterior olfactory nucleus, retrobulbar (AONrb), anterior olfactory nucleus, cortical anterior (AONca) and anterior olfactory nucleus, cortical posterior (AONcp), respectively) in PD and control cases. This is the first report describing variable α-synuclein susceptibility among specific neuronal populations in PD.

## Materials and Methods

### Human Brain Tissue

Specimens from four neuropathologically diagnosed cases of PD (stages IV and V) and four age-matched controls were examined in this study (Table 1). Tissue blocks were provided by IDIBAPS (Barcelona), BTCIEN (Madrid), and BIOBANC-MUR (Murcia) Biobanks. Experimental procedures were approved by the Ethical Committee of Clinical Research at Ciudad Real University Hospital (SAF2016-75768-R).

**Table 1:** Demographic and clinic-pathological features of the individuals used in this study.

Diagnosis	Sex	Age	Brain weight (g)	Stage
PD	M	67	1425	Braak IV-V
PD	M	71	1275	Braak V/AD Braak III
PD	F	69	1065	Braak IV-V/tau Braak III
PD	M	55	790	Braak V/AD Braak III
C	M	69	1451	
C	M	87	1000	
C	M	59	1400	
C	M	72	1600	

*C, control; F, female; M, male; PD, Parkinson’s disease*.

## Histology

Tissue was stored in 4% paraformaldehyde for 45 days and cryoprotected by immersion in 2% dimethyl sulfoxide (DMSO) and 10% glycerol for 48 h, followed by 2% DMSO and 20% glycerol for a further 48 h. Using a freezing sliding microtome, 10 series of 50-μm sections were obtained. One of the series was Nissl-stained, while the second was used for immunohistochemistry against α-synuclein; the third and fourth series were used for immunofluorescence analysis.

Two different immunofluorescent experiments were carried out. Sections were incubated with primary antibodies against α-synuclein, parvalbumin and calretinin, or α-synuclein, calbindin and somatostatin, followed by Alexa Fluor-conjugated secondary antibodies (Table 2) according to previous protocols (Ubeda-Bañon et al., 2010; Flores-Cuadrado et al., 2017).

**Table 2:** Antibodies used in the present study.

Antigen	Dilution	Species	Secondary antibody (1/200)
Calbindin D-28k. Swant (Marly, Switzerland)	1:1000	Rabbit polyclonal	Alexa Fluor^®^ 488 anti-rabbit. Molecular Probes (Eugene, OR, USA)
Calretinin CR-769. Swant	1:1000	Rabbit polyclonal	Alexa Fluor^®^ 488 anti-rabbit. Molecular Probes
Parvalbumin PVG-214. Swant	1:1000	Goat polyclonal	Alexa Fluor^®^ 647 anti-goat. Molecular Probes
Somatostatin D-20. Santa Cruz (Dallas, TX, USA)	1:500	Goat polyclonal	Alexa Fluor^®^ 647 anti-goat. Molecular Probes
α-synuclein. Novocastra (Newcastle, UK)	1:20	Mouse monoclonal	Alexa Fluor^®^ 555 anti-mouse. Molecular Probes

### Quantification

**Figure 1:**
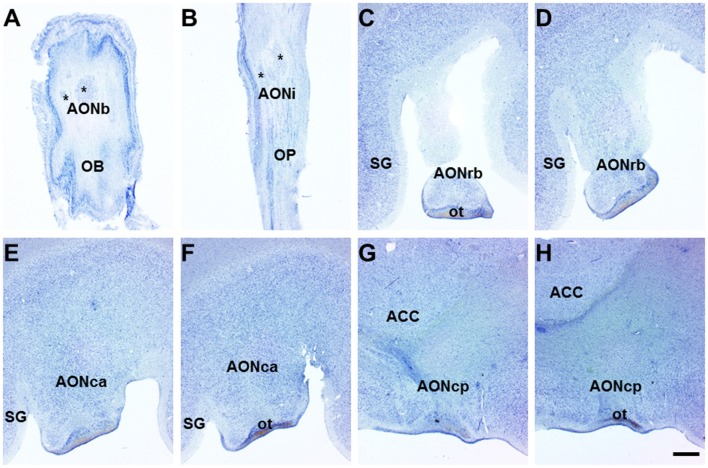
Horizontal (olfactory bulb (OB) and olfactory peduncle (OP)) and coronal (frontal lobe) sections of the human brain. Nissl-stained horizontal sections of the human OB and OP show bulbar **(A)** and intrapeduncular **(B)** anterior olfactory nucleus (AON) showing diverse components (asterisks). In coronal sections of the frontal lobe, the retrobulbar **(C,D)**, cortical anterior **(E,F)** and cortical posterior **(G,H)** AON are distinguishable. Scale bar, 1300 μm. For abbreviations, see list.

**Figure 2:**
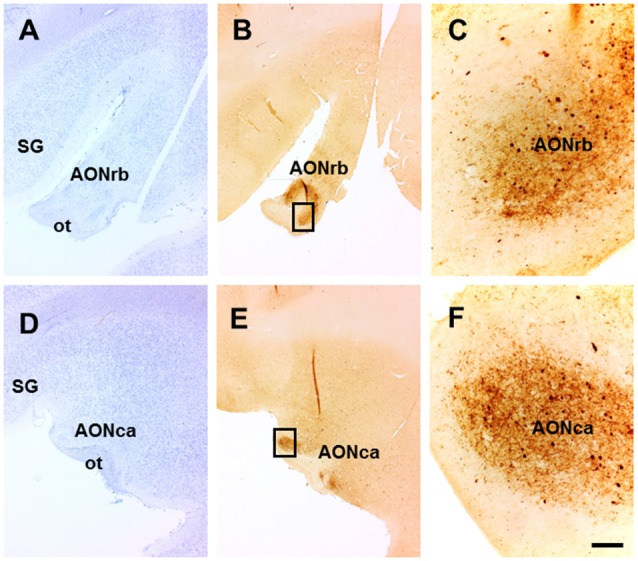
Nissl staining **(A,D)** and the parallel coronal sections showing α-synuclein immunoreactivity **(B,C,E,F)** of the anterior olfactory nucleus, retrobulbar (AONrb) **(A–C)** and anterior olfactory nucleus, cortical anterior (AONca) **(D–F)** divisions of the human AON. **(C,F)** correspond to framed area shown in **(B,E)**, respectively. Scale bar, for **(A,B,D,E)** 1300 μm, for **(C,F)** 130 μm. For abbreviations, see list.

Three divisions of the AON—the AONrb, and the AONca and AONcp divisions (Figure 1)—which were identified in parallel Nissl-stained sections at approximately 12.5, 10.0 and 7.5 mm from Bregma (Mai et al., 2008), were analyzed in each case (*n* = 8; Figure 2).

Using LSM 800 confocal microscope (Zeiss, Jena, Germany) a total of 192 images at 20× magnifications were acquired: four random images in each division (*n* = 3) of each case (*n* = 8) of each immunofluorescent staining combination (*n* = 2). In every image, four independent channels were acquired according to the three secondary antibodies used (Table 2), including DAPI.

For analysis, ZEN (Zeiss, black edition 10.0) and ImageJ software (National Institutes of Health, Bethesda, MD, USA, 1.47v) were used. In order to estimate changes in the expression of markers in neuronal cell bodies alone, changes in expression of markers in neuronal cell bodies and neurites, and in co-localization of markers with α-synucein, three kinds of parameters were measured: cells/mm^2^, % of area fraction and % of co-localization, respectively.

For estimating the number of positive cell bodies (cells/mm^2^) of calcium binding proteins and somatostatin, the images were analyzed with ImageJ using a protocol for the automated counting of stained cells as previously reported (Flores-Cuadrado et al., 2015). The corresponding channel of the marker analyzed was selected in every image and the images were converted to a binary 8-bit grayscale and a histogram was generated. The mode was multiplied by 0.6 (60%) to obtain the threshold for distinguishing specific cell labeling from background. The thresholds were: 71 for somatostatin-positive cells, 30 for calbindin-positive cells, 40 for calretinin-positive cells and 90 for parvalbumin-positive cells.

In order to quantify the total expression of markers, including cell bodies and neurites, % of area fraction was analyzed as previously described (Saiz-Sanchez et al., 2012). Using the the same images as above, the labeled area was compared to un-labeled tissue as an automatic parameter of ImageJ.

For co-localization of different neural and pathological markers, ZEN and ImageJ software was used. Using two channels from every 2D image, the total number of cells expressing a neural marker was compared to the number of cells that also co-expressed α-synuclein. In this way, co-localization as a percentage of total cells was obtained.

Data were analyzed using GraphPad Prism software (GraphPad Inc., San Diego, CA, USA, v.6). The normality of the samples (*P* > 0.05) was evaluated with the Kolmogorov–Smirnov test and group means were compared with the Mann–Whitney *U* test (non-parametric data). Data are expressed as mean ± standard error of the mean to estimate the reliability of the mean. Differences were considered statistically significant at *P* < 0.05.

## Results

The human AON is a complex and multiple-portion structure that is poorly characterized. Classical descriptions include several subdivisions along the olfactory system (Crosby and Humprey, 1941) that have been renamed as the bulbar, intrapeduncular, retrobulbar, cortical anterior and cortical posterior divisions (Ubeda-Bañon et al., 2010). Horizontal sections of the human OB (Figure 1A) and olfactory peduncle (OP; Figure 1B) showed several components of the anterior olfactory nucleus, bulbar (AONb; Figure 1A, asterisks) and anterior olfactory nucleus, intrapeduncular (AONi) AON (Figure 1B, asterisks). In coronal sections, the AONrb displayed a typical horseshoe form (Figures 1C,D) that once incorporated to the basal prosencephalon, merged into the AONca (Figures 1E,F), and caudally, into the AONcp (Figures 1G,H).

Using sequential Nissl-stained sections, the AONrb (Figure 2A), AONca (Figure 2D), and AONcp (not shown) were analyzed. Immunohistochemical analysis of α-synuclein expression revealed specific immunoreactivity in different AON divisions includig the AONrb (Figure 2B), AONca (Figure 2E) and AONcp (not shown). High-power images demonstrated the preferential labeling of Lewy bodies and neurites in the AONrb and AONca divisions, respectively (Figures 2C,F).

**Figure 3:**
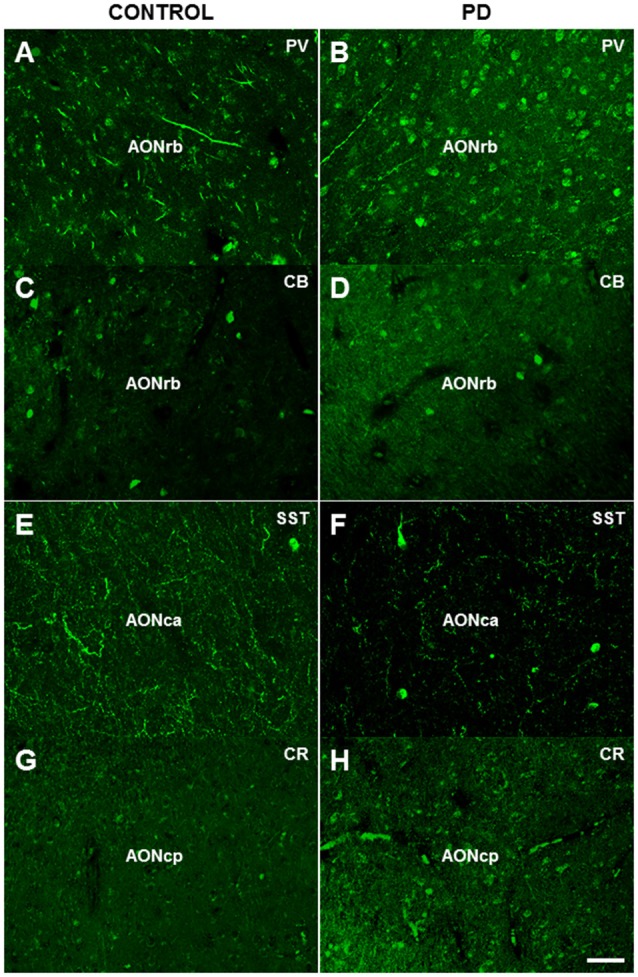
Immunofluorescence analysis of parvalbumin **(A,B)**, calbindin **(C,D)**, somatostatin **(E,F)** and calretinin **(G,H)** expression in different divisions of the human AON. Scale bar, 50 μm **(A–H)**. For abbreviations, see list.

**Figure 4:**
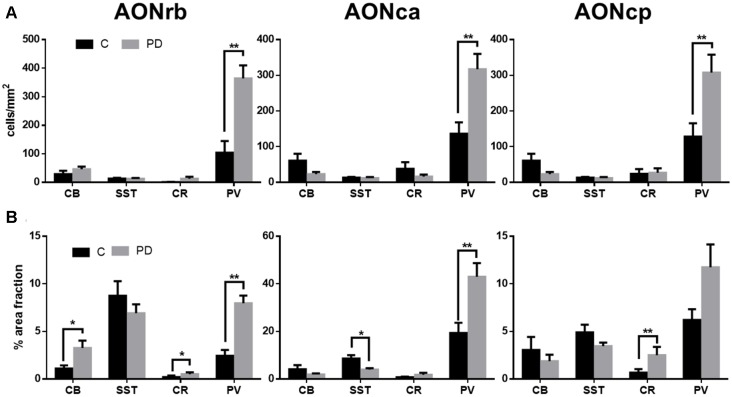
Graphs show cells/mm^2^ **(A)** and % of area fraction **(B)** expression of calbindin, somatostatin, calretinin and parvalbumin in the different portions of the AON in Parkinson’s disease (PD) and control (C) cases. *p < 0.05, **p < 0.01.

**Figure 5:**
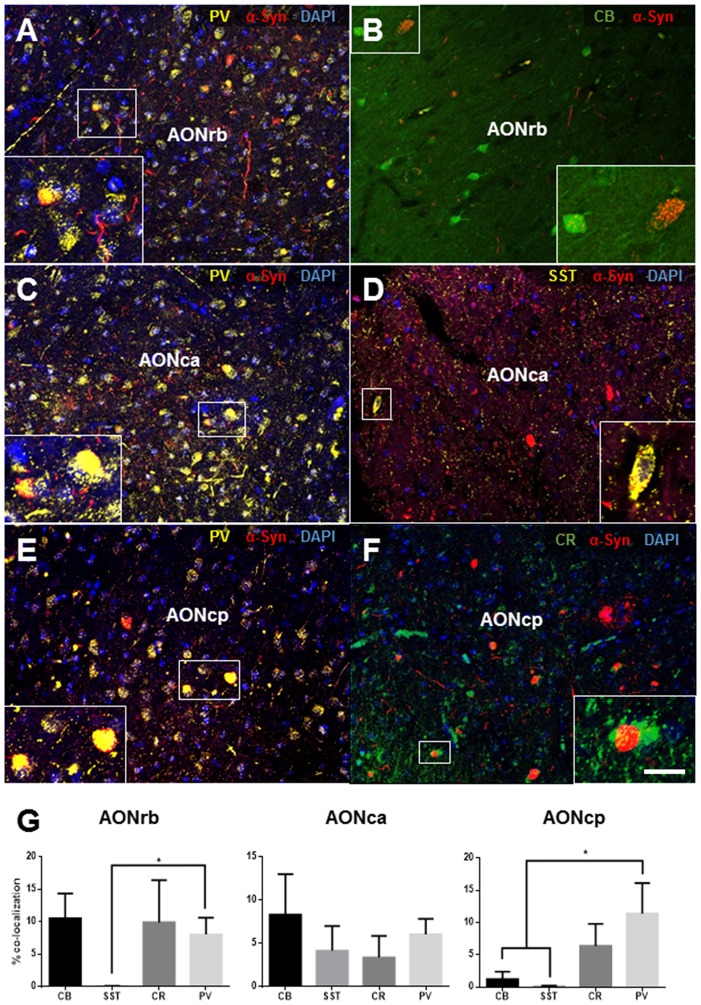
Immunofluorescence analysis of parvalbumin **(A,C,E)**, calbindin **(B)**, somatostatin **(E)** and calretinin **(F)** expression and their co-localization with α-synuclein in different divisions of the human AON. Percent is referred to total number of cells counted that express a neural marker and the fraction of which co-express α-synuclein. Percent of co-localization of α-synuclein with the interneuron markers indicated **(G)** in the AONrb, AONca and anterior olfactory nucleus, cortical posterior (AONcp) divisions of the AON. Scale bar, 50 μm **(A–F)**; 21.25 μm in frame **(A)**, 24.53 μm in frame **(B)**, 16.72 μm in frame **(C)**, 10.31 μm in frame **(D)**, 16.43 μm in frame **(E)**, 12.5 μm in frame **(F)**. For abbreviations, see list. *p < 0.05.

Fluorescent labeling (Figures 3, 5) was analyzed using three parameters: (1) we compared the respective neuronal markers in the neuronal cell bodies (cell/mm^2^) located in the divisions of the AON of PD and control cases (Figure 4B); (2) we compared the expression of these markers in the neuronal cell bodies and neurites (% of area fraction) in the divisions of the AON of PD and control cases (Figure 4A); (3) we analyzed the co-localization of these neuronal markers with α-synuclein (Figure 5).

Immunofluorescent analyses revealed significantly higher expression of parvalbumin in the neuronal cell bodies (cell/mm^2^) in all of the AON divisions of PD cases compared with controls: the AONrb (*P* = 0.001; Figures 3A,B, 4A), AONca (*P* = 0.001; Figure 4A) and the AONpc (*P* = 0.087; Figure 4A). No significant differences were found in the expression of calbindin, somatostatin, or calretinin in the above regions between the two goups (Figures 3C–H, 4A).

There was significanlty higher expression of calbindin (*P* = 0.0178; Figures 3C,D, 4B), calretinin (*P* = 0.0366; Figure 4B) and parvalbumin (*P* = 0.0001; Figures 3A,B, 4B) in the cell bodies and neurites (% of area fraction) of neurons in the AONrb of PD patients compared with controls. There was also significantly higher expression of parvalbumin in the neurites (Figure 4B) of neurons in the AONca of PD cases compared with controls (*P* = 0.0031; Figure 4B). In contrast, somatostatin expression was significantly downregulated in the AONac of PD cases (*P* = 0.0113; Figures 3E,F, 4B). Finally, calretinin expression was significantly higher in the cell bodies plus neurites of neurons in the AONpc of PD patients (*P* = 0.0096; Figures 3G,H, 4B).

Co-localization of α-synuclein and the above neural markers in PD cases was analyzed to investigate involvement of α-synucleinopathy among different neuronal subtypes, and is summarized in Figure 5. Examples of co-localization of α-synuclein in the AONrb (Figures 5A,B), the AONca (Figures 5C,D), and the AONpc (Figures 5E,F) with calretenin, calbindin, parvalbumin and somatistatin are shown. Analyses of the percentage of cells where co-localization occurred revealed that neurons expressing the calcium-binding proteins (calretinin, calbindin and parvalbumin) had the highest percentages of α-synuclein co-localization. Somatostatin/α-synuclein-positive cells were rare, although there were higher percentages in the AONca (Figures 5D,G).

Significant differences were also observed between somatostatin and parvalbumin expression in the AONrb of PD cases (*P* = 0.0113; Figure 5G) and between somatostatin and parvalbumin expression and calbindin and paravalbumin expression in the AONcp (*P* = 0.0124; Figure 5G).

## Discussion

We found that increased expression of calcium-binding proteins (calretinin, calbindin and parvalbumin), particularly parvalbumin, correlated with α-synuclein co-localization in the different divisions of the AON. In contrast, the decreased neuritic expression of somatostatin in the AONca also led to decreased α-synuclein co-localization. Varied calbindin expression (% of area fraction) and calbindin/α-synuclein co-localization among the divisions of the AON were also found. Regarding calretinin expression, we did not observe differences in any of the AON divisions in cells/mm^2^, but we did find significant increases in the % of area fraction in both the AONrb and AONcp. This contrast to recent data reporting significant reductions in the number of calretinin-positive periglomerular and granular cells in the OB of PD cases compared to controls (Cave et al., 2016). Data from this experiment support the idea for differential regional α-synuclein involvement; and therefore, we believe the divisions of the AON to be diverse and thus need to be independently analyzed.

There are several limitations to this study. First and foremost, the interpretation of the data obtained in this study is limited by the number of cases available. Future studies including larger number of cases are needed.

This report is the first study comparing both the expression of markers of different neuronal populations in the AONrb, AONca, and AONcp of PD cases and controls as well as α-synuclein distribution in these respective areas. Previous reports on α-synuclein distribution in the OB focused on the AONb and/or AONi (Del Tredici et al., 2002; Braak et al., 2003a,b; Attems et al., 2014; Del Tredici and Braak, 2016; Braak and Del Tredici, 2017) and for the most part, did not include co-localization with neural markers (Sengoku et al., 2008).

In this context, it is especially interesting to investigate the differential vulnerability of interneuron populations in the AON since this structure is implicated in hyposmia (Hawkes and Shephard, 1993; Jellinger, 2009) and shows early and preferential involvement in α-synucleinopathy (Sengoku et al., 2008; Ubeda-Bañon et al., 2010; Attems et al., 2014); suggesting that it is an important structure in PD etiology (Ubeda-Bañon et al., 2014). The early and preferential involvement of the AON in PD could be due to the multiple interconnections of this structure with various other regions of the brain as shown in rodents (Brunjes et al., 2005). Indeed, a direct projection from the substantia nigra to the OB has recently been described (Höglinger et al., 2015). Therefore, as the AON in the human brain is composed of at least five different divisions (Ubeda-Bañon et al., 2014; Figure 1), each of these needs to be further characterized in order to establish accurate analysis.

Overall, our results show high levels of calcium-binding proteins—specfically of parvalbumin—in the AON and reduced neuritic somatostatin expression in the AONca; in contrast to what is observed in the substantia nigra (Hardman et al., 1996).

In the human amygdala, we reported decreased expression of somatostatin in PD cases (Flores-Cuadrado et al., 2017), but, in contrast to the current data, we also found decreased expression of parvalbumin (Flores-Cuadrado et al., 2017). This difference could be due to the fact that the number of parvalbumin-positive cells/mm^2^ is 10 times higher in the AON (Figure 4) as compared to the human amygdala (Flores-Cuadrado et al., 2017). Also, the intrinsic dendrites vs. somatostatin-positive axons from distal sites cannot be discarded (Lepousez et al., 2010). Interestingly, calcium-binding proteins, but rarely somatostatin, co-localized with α-synuclein, which was in agreement with previous studies (Flores-Cuadrado et al., 2016, 2017).

In Alzheimer’s disease, somatostatin expression is decreased by 50% in the AONca and co-localizes with amyloid β (Saiz-Sanchez et al., 2010), whereas parvalbumin and somatostatin levels are up- and downregulated, respectively, in the piriform cortex (Saiz-Sanchez et al., 2015, 2016). Common patterns of differential vulnerability among different interneuron populations due to distinct proteinopathies (Epelbaum et al., 2009) deserves additional analysis in structures like the AON, which is involved early and preferentially.

Our results suggest a potential differential vulnerability that may be due to α-synucleinopathy among interneurons populations in different divisions of the human AON. It is unclear to what extent cells expressing calcium-binding proteins can buffer the pathological effects of α-synuclein and somatostatin-positive neurons are induced to change their cell body size and/or dendritic expression by non-aggregated α-synuclein isoforms; these are questions that will be addressed in future studies.

## Author Contributions

IU-B, AF-C, DS-S and AM-M: study design, discussion and writing. IU-B, AF-C and DS-S: tissue section. IU-B and AF-C: immunohistochemistry and data analysis. IU-B and DS-S: image capture. IU-B, AF-C, DS-S and AM-M: discussion and writing.

## Conflict of Interest Statement

The authors declare that the research was conducted in the absence of any commercial or financial relationships that could be construed as a potential conflict of interest.

## Abbreviations

ACC: nucleus accumbens
AON: anterior olfactory nucleus
AONb: anterior olfactory nucleus, bulbar
AONca: anterior olfactory nucleus, cortical anterior
AONcp: anterior olfactory nucleus, cortical posterior
AONi: anterior olfactory nucleus, intrapeduncular
AONrb: anterior olfactory nucleus, retrobulbar
C: control
CB: calbindin
CR: calretinin
DMSO: dimethyl sulfoxide
OB: olfactory bulb
OP: olfactory peduncle
ot: olfactory tract
PD: Parkinson’s disease
PV: parvalbumin
REM: rapid eye movement
SG: straight gyrus
SST: somatostatin
α-syn: α-synuclein.
